# Valuing breeders' preferences in the conservation of the Koundoum sheep in Niger by multi-attribute analysis

**DOI:** 10.5194/aab-62-537-2019

**Published:** 2019-09-09

**Authors:** Issa Hamadou, Nassim Moula, Seyni Siddo, Moumouni Issa, Hamani Marichatou, Pascal Leroy, Nicolas Antoine-Moussiaux

**Affiliations:** 1 Department of Animal Production, National Institute of Agronomic Research of Niger, P.O. Box 429, Niamey, Niger; 2 Fundamental and Applied Research for Animals & Health (FARAH), Sustainable Animal Production, Faculty of Veterinary Medicine, University of Liège, 4000 Liège, Belgium; 3 Tropical Veterinary Institute, Faculty of Veterinary Medicine, University of Liège, Quartier Vallée 2, Avenue de Cureghem 6, building B43, 4000 Liège, Belgium; 4 Department of Animal Production, Faculty of Agronomy, Abdou Moumouni University, P.O. Box 10 960, Niamey, Niger

## Abstract

This study characterises farmer's preferences for breeding rams and tackles their willingness to contribute to the Koundoum sheep conservation programme through their quantified appreciation of the main phenotypic features of the sheep breed in the region. The Koundoum is the main wool sheep of Niger and shows a remarkable adaptation to the environment of the Niger River valley. In Tillabéri region, i.e. the Koundoum sheep's area of origin, the proportional piling tool is first used in 11 focus group discussions of breeders to determine the main selection criteria of breeding rams. The multi-attribute analysis method is then applied with 168 sheep owners. The econometric estimation of the utility function of breeders is conducted with a conditional logit model and the marginal willingness to pay is calculated. The results reveal a strong rejection by the breeders of characteristics like wool and black-coloured coat and thus shows the poor acceptability of an in situ conservation programme. Few breeders with a particular concern for the breed's conservation for cultural motives may nevertheless join such a conservation programme that should mainly be based on ex situ strategies.

## 1 Introduction

In developing countries the livestock species play very important economic, social and cultural roles or functions for rural households once they contribute to improving the wellbeing and income of the farm family. Livestock helps with food supply, family nutrition, family income, asset savings, soil productivity, livelihoods, transport, agricultural traction, agricultural diversification and sustainable agricultural production, family and community employment, ritual purposes, and social status (Moyo and Swanepoel, 2010).

Mainly in sheep and cattle, the diffusion of breeds showing high production abilities and the homogenisation of the production systems go along with a neglect of more resilient indigenous breeds. This substitution between breeds and the uncoordinated use of crossbreeding lead to an erosion of animal genetic resources in Niger and West Africa as in the rest of the world (Rege and Gibson, 2003).

In Africa, livestock research has mainly focused in the past on cattle at the expense of small ruminants (Bidjeh et al., 1991). However, the rusticity and shorter reproduction cycle of sheep and goats, allowing for the rapid restocking after major droughts, justify the present increase of interest in these species in Africa (Bloch and Diallo, 1991). Also, the lack of seasonality in oestrus manifestations in ewes in the tropics is a dynamic advantage in this regard (Hamadou et al., 2015b). The importance of sheep in Muslim traditions further explains the present dynamism of these markets across Sahel countries and the intensification of sheep husbandry practices accompanying urbanisation. The sustainable development of sheep production in the highly variable environmental conditions of Sahel countries needs to be based on the wide genetic diversity that is indeed observed in sheep in these countries (Shrestha et al., 2010). However, this genetic diversity is currently declining, mainly due to the socio-economic and cultural processes mentioned here above, motivating the neglect of several indigenous breeds (Sechi et al., 2005).

In terms of breed survival, rapid change may mean that a breed's existing role disappears rapidly and that it declines towards extinction before new roles for it can emerge or national authorities recognise the threat and take action to promote its conservation (FA0, 2015). Indeed, the majority of breeders practice a reasoned choice of breeding stock according to criteria in accordance with their production objectives. In sheep breeding, therefore, growth criteria may take priority over ones of environmental adaptation. In particular, the use of crossbreeding with exotic breeds may be a concern because of the lack of adaptation of these animals to the local production environment and the irremediable loss of indigenous purebred genetic resources with the spread of this practice (Wollny, 2003). In Niger, the Koundoum sheep is the country's main wool breed and is adapted to the damp environment of the Niger River valley. According to Toubo (1975), Koundoum sheep are exclusively raised in the islands and on the river banks during flood periods, from the boarder of Mali to Niamey; the koundoum sheep habitat has a length of 200 km, and a width that never exceeds 20 km.

The Koundoum breed is described by Toubo (1975), as a medium-sized animal with slightly convex profile. The body is covered with wool; the head, the belly and the limbs are naked. The fleece is black and white with black spots on the head (Hamadou et al., 2015a). The horns are smaller and often absent in the female. In the male, the horns are highly developed, prismatic and directed backwards (Meyer et al., 2004). The ears are long, wide, thick and drooping. Its meat production performances are lower in comparison to Fulani sheep, with a mean adult weight of 30 and 25 kg in males and females, respectively, and a carcass percentage of 40 %. Despite this low yield, the meat of Koundoum sheep is reported to be lean and renowned for its taste and tenderness (Hamadou et al., 2015a).

The neglect of this breed in favour of taller and heavier sheep breeds such as Fulani sheep (Toubo, 1975) and the uncoordinated practice of crossbreeding are leading to a drastic reduction in the Koundoum population. Without a conservation programme, this breed is doomed to extinction in the medium term.

In 2010, the University of Niamey initiated a conservation programme for this breed in the framework of a national conservation plan of animal genetic resources.

An efficient way to conserve genetic resources is often to help farmers improve their indigenous breeds and to use them (Planchenault and Boutonnet, 1997). To evaluate the opportunity for such in situ conservation schemes and design them, it is necessary to understand the breeders' preferences regarding their breeding decisions (Jabar et al., 1999; Tada et al., 2013; Bayou et al., 2014). Multi-attribute choice experiments may be used to evaluate the preferences of breeders, particularly expressed as a willingness to pay or to receive compensation for the various levels of the characteristics of a proposed ram. A choice experiment is a quantitative technique that determines individual preferences by submitting multiple virtual choice tasks to interviewees (Hanley et al., 1998; Mangham et al., 2009). These methods have been widely used to estimate the willingness of respondents to pay or receive compensation for animal genetic resources in different breeding systems, mainly in developing countries (Ruto et al., 2008; Zander and Drucker, 2008; Tada et al., 2013).

This study applies the multi-attribute choice experiment to the case of Koundoum sheep appreciation by breeders in Tillabéri, Niger, that is the area of origin of this breed. It aims to clarify the valuation of different attributes of the sheep according to a willingness to pay and willingness to accept compensation in order to better understand the feasibility of a subsidised in situ conservation scheme and shape conservation messages to be diffused among breeders. According to Drucker et al. (2001), the payment valuation method based on willingness to pay (WTP) or willingness to accept (WTA) for conservation is a promising option for biodiversity valuation in general because it is the only way to elicit non-use values directly. In this aspect, the potential for information provision and exchange during the survey process offers scope to experiment with respondent knowledge and understanding of biodiversity. This method can be used as a surrogate referendum for determining conservation priorities based on public preferences.

## 2 Materials and methods

### 2.1 Study area

**Figure 1:**
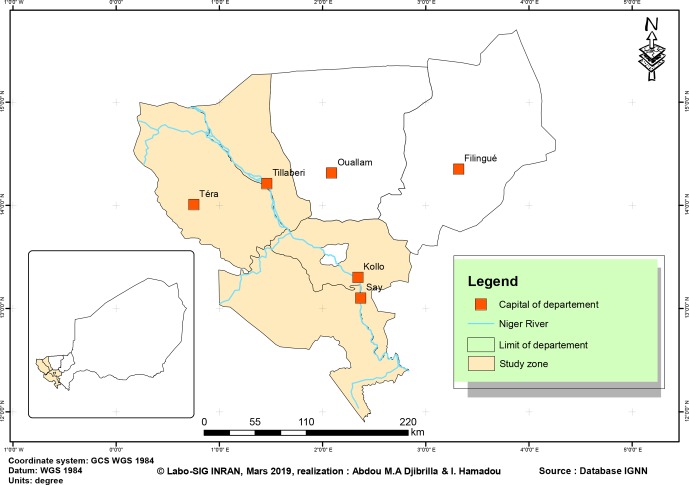
Map of Tillabéri area; source: INRAN Labo SIG Database.

The study was conducted from September 2012 to February 2013, in four departments of the administrative region of Tillabéri (Niger), i.e. Kollo, Say, Téra and Tillabéri (Fig. 1). This region is located at the extreme west of the country, in the Niger River valley. Economic activities there are livestock, agriculture, forestry and fishing. From June to September, rain-fed agriculture is practised (mainly pearl millet). From October to March, the period corresponding to the dry season, market gardening is practised. Households mostly also keep animals. Three animal production systems are practised: extensive sylvo-pastoral, semi-intensive agro-pastoral and intensive agro-pastoral systems.

### 2.2 Participatory survey on breeding ram appreciation criteria

#### Sampling of focus groups

The identification of appreciation criteria was undertaken in 11 focus groups with 10 persons each (including 4 focus groups in Tillabéri, 3 in Kollo, 2 in Say and 2 in Téra). The sheep keepers were selected by snowball sampling on basis of first interviewees, randomly selected among a list provided by local authorities. With each focus group, an open discussion was first led about the appreciation criteria of breeding rams. The criteria were listed, written and represented by symbols on paper (for illiterate participants). To each criterion a relative importance was then assigned through proportional piling, using 100 counters. Proportional piling is defined as a technique used to get people to express the different importance of issues, events and things to a particular community.

The consensus was sought through an iterative process and written notes were taken about ongoing discussions.

This preliminary work allowed taking account of the views of sheep breeders on the choice of the breeding ram traits to be included as multilevel attributes in the choice experiments.

### 2.3 Multi-attribute analysis of selection criteria

#### 2.3.1 Identification of attributes, levels and building of comparison profiles

**Figure 2:**
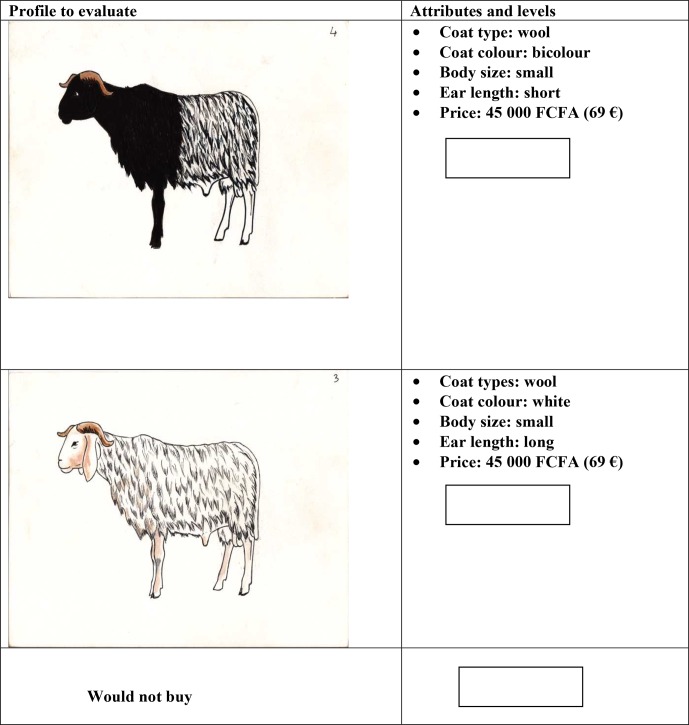
Example of pairwise comparison choice card source, illustrated by a local artist. FCFA: Franc of the African Financial Community.

Four attributes with two to three levels each were retained to establish the stated preference protocol. The selection of attributes was made according to citation rate and proportional piling scores. A price attribute was established on basis of local market information. Three levels were determined, i.e. EUR 69 (FCFA 45 000), EUR 53 (FCFA 35 000) and EUR 38 (FCFA 25 000), which represent, respectively, the mean prices of young Fulani rams, crossbred ram and Koundoum ram according to local market information resulting from discussion with sellers and buyers (EUR 1 = FCFA 655 957). A fractional factorial design verifying the absence of correlation between attributes levels was applied in XLSTAT 2013 software using the D-optimal algorithm to generate 16 rams' profiles. Then 20 pairs of choices consisting of two opposite profiles were selected with the same software. These profiles were illustrated by a local artist (Fig. 2).

#### 2.3.2 Stated preference survey: sampling and interview

The criterion for inclusion in the stated preference survey was to be a sheep owner. In each face-to-face interview in the local language Djerma, the 20 pairs of profiles were proposed to the interviewee, asking him which animal he would buy. For each pair, the interviewee had the possibility to opt out, i.e. to assert that none of the two profiles were acceptable to him.

#### 2.3.3 Statistical analysis and estimation of the willingness to pay

Econometric analysis of stated preferences was performed with the R software (R 3.0.1, package *survival*, package *support.Ces*). The price variable was expressed in euros for statistical analysis. A conditional logit model estimated the utility coefficients related to the various attributes of rams and the willingness of breeders to pay or receive compensation for the various levels of these attributes. The conditional logit model is based on the random utility function

1 $$U_{\mathrm{in}}=V_{\mathrm{in}}+\varepsilon_{\mathrm{in}},$$

with $U_{\mathrm{in}}$ the utility of individual n for scenario i, $V_{\mathrm{in}}$ the deterministic component of utility and $\varepsilon_{\mathrm{in}}$ an unobservable component of utility, considered as a random component, which is the utility contributed by attributes unobserved by the analyst.

The probability of choosing one of these ram i profiles is

2 $$\mathrm{Pr}\{i\;\mathrm{is}\;\mathrm{chosen}\}=\mathrm{Pr}\left\{V_{ni}+\varepsilon_{ni}\geq V_{nj}+\varepsilon_{nj};\;\mathrm{for}\;\mathrm{all}\;j\in C_{i}\right\},$$

where C is the set of choice for the breeder n ($C_{n}=\{1$, 2, 3}), the choice 3 = “no choice”). For each individual n, the utility provided by the choice of scenario i is in the form $V_{\mathrm{in}}=\alpha_{i}+\sum \beta_{k}x_{ik}$, with $\alpha_{i}$ a specific constant to scenario i (ASC), and $\beta_{k}$ coefficients to estimate for the k attributes whose values in the scenario i are represented by the $x_{ik}$.

The willingness to pay corresponds to a monetary conversion of utility coefficients of each attribute's level, according to the method described by Tada et al. (2013). The willingness to pay for a level l of an attribute k is calculated as follows: WTP${}_{kl}=-\beta_{kl}/\beta_{\mathrm{EUR}}$, with $\beta_{kl}$ as defined earlier and $\beta_{\mathrm{EUR}}$ being the utility coefficient of the monetary unit (EUR).

The relative importance of an attribute k was calculated as RI${}_{k}=100\times (\beta_{kl\_\max}-\beta_{kl\_\min})/\sum (\beta_{kl\_\max}-\beta_{kl\_\min})$, where $\beta_{kl\_\max}$ and $\beta_{kl\_\min}$ are, respectively, the maximal and minimal utility coefficient among the levels l of an attribute k.

## 3 Results

### 3.1 Appreciation criteria for breeding rams

Nine breeding criteria were collected through the 11 focus group discussions (Table 1). The four main criteria are coat colour, which has the largest proportional piling score (25.6 %), body size (24.5 %), type of coat (21.6 %) and ear length (14.9 %). Thus, the attributes and levels that were included in the stated preference survey are as follows: coat type (bristles or wool), coat colour (white, bicolour or black), body size (large or small) and ear length (long or short).

**Table 1:** Results of proportional piling (PP score) regarding breeding criteria in 11 focus groups of sheep breeders in Tillabéri region, Niger.

Criteria	Citation	PP score	Median	Min	Max
	rate (%)	(%)			
Coat type	100	21.6	21	11	39
Coat colour	100	25.6	27	8	43
Body size	100	24.5	24	10	51
Ear length	91.0	14.9	15	0	33
Wattles	18.2	1.6	0	0	11
Tail length	55.0	6.7	0	0	17
Testicle size	18.2	1.0	6	0	6
Head size	9.1	1.1	0	0	12
Horn type	36.4	3.0	0	0	11
Total PP score (%)		100.0			

Note: the median, min and max were assessed on the PP score.

**Table 2:** Utility coefficients and willingness to pay estimated for breeding ram traits in Niger.

Attributes	Levels	Marginal utility	MWTP	CI 95 % (EUR)
			(EUR)	
Coat type	Bristles	$1.63\pm 0.06^{***}$	226	[132.6, 696.0]
	Wool	0	–	–
Coat colour	White	$0.05\pm 0.07^{\mathrm{ns}}$	7	[-15.4, 42.9]
	Black	$-1.23\pm 0.08^{***}$	-172	[-552.5, -96.8]
	Bicolour	0	–	–
Body size	Large	$0.33\pm 0.07^{***}$	46	[18.8, 156.1]
	Small	0	–	–
Ear length	Long	$0.78\pm 0.06^{***}$	109	[60.6, 345.6]
	Short	0	–	–
Price	–	$-0.007\pm 0.003^{**}$	–	–

MWTP: mean willingness to pay. CI: confidence interval. Significance codes: ${}^{***}$ p≤0.001; ${}^{**}$ p≤0.01; ns – not significant.

### 3.2 Conditional logit analysis and willingness-to-pay calculation

A total of 168 sheep owners participated in the stated preference survey. The results of the conditional logit show a pseudo-$R^{2}$ of 0.162 and a positive but not significant coefficient associated with the constant $\alpha_{i}$ (p>0.5). The utility coefficients estimated for all attribute levels are presented in Table 2. For each attribute, a level is defined as a reference and the coefficients of the other levels represent the value acquired through a change from the reference level to the level considered. The utility coefficient of the monetary unit is negative (-0.0073) and highly significant (p<0.001). The appreciation of white coat colour is positive but statistically not different from the reference bicolour coat. Black coat colour shows a negative and statistically highly significant utility coefficient, thus depreciating a ram's value compared to other coat colours (p<0.001). All other utility coefficients are positive and statistically highly significant (p<0.001).

As expressed in terms of willingness to pay, these appreciated attribute levels are thus long ears (EUR 109) with a CI 95 % of [60.6, 345.6], bristles (EUR 226) with a CI 95 % of [132.6, 696.0] and large body size (EUR 46) with a CI 95 % of [18.8, 156.1]. For black-coloured coats we have a negative willingness to pay of (-172) with a CI 95 % of [-552.5, -96.8]. In this case, instead of willingness to pay we talk of willingness to accept payment. The willingness to accept compensation for black-coloured coats is EUR 172. The attribute “coat type” shows the highest relative importance in the decision making with 40 %. The attribute “coat colour” comes second with 31 % and then “ear length” and “body size”, with 16 % and 13 %, respectively (results not shown).

## 4 Discussion

### 4.1 Methodology

The administrative region of Tillabéri has three animal production systems, i.e. extensive sylvo-pastoral, semi-intensive agro-pastoral and intensive agro-pastoral systems, with a general trend in shift from pastoral systems to agro-pastoral systems. In general, in sub-Saharan Africa, for example, Thornton et al. (2002) predict a substantial change in pastoral systems and in agro-pastoral systems over the next 50 years.

This study identifies the criteria to include in the stated preference protocol through participatory methods, i.e. focus group discussions and proportional piling. Indeed, taking account of the views of breeders at all steps of the design and implementation of animal genetic resource conservation programmes is essential to promote the appropriation and sustainability of this programme (Wollny, 2003). This step of the study allowed stimulating the interest of the breeders for the research and its subject, the Koundoum sheep, and eased their further participation in the process. Similar approaches have been taken in Ethiopia, for example, to define local breeding objectives and preferred characteristics in goats (Gebreyesus et al., 2013) or to characterise the Simien sheep breed (Melaku et al., 2012).

The multi-attribute analysis protocol applied here makes use of an opt-out choice. This option allows consumers to choose none of the alternatives when those are not deemed interesting (Ohannessian, 2008). This non-choice makes the decision of choice more realistic since the respondent is not forced to state an appreciation of unacceptable products. However, one might also opt out as a result of too a high similarity of interests between two acceptable products. The motives to opt out may therefore be clarified with the respondent during the interview.

The present sample size of breeders is in conformity with the standard reported by Omondi et al. (2008), who indicate a minimum size of 100 households. Moreover, the positive and non-significant value of the coefficient associated with the constant obtained in the conditional logit confirms the relevance of the reference profile. There is then no bias due to reference that may affect results (Scott, 2001). The pseudo-$R^{2}$ value (0.162) obtained in the conditional logit model indicates an acceptable estimate of the model, referring to an acceptance threshold of 0.1 (Roessler et al., 2008).

### 4.2 Appreciation criteria for breeding rams

Among the nine selection criteria, only three are found in the results of all the 11 focus groups, i.e. coat type, coat colour and body size. A fourth attribute, ear length, was also included in the protocol, being found in 10 focus groups. The concern is here to limit the number of attributes and levels, in order to limit the number of profiles to be proposed to respondents' choice and thus limit the complexity of the submitted task (Louviere et al., 2010). The body size and the coat colour are classical criteria in traditional breeding systems in Africa, being used also, for example, in Ankole cattle in Uganda (Kugonza et al., 2012).

Nevertheless, less cited criteria might also show significant importance in respect to further developments of a breeding or conservation programme. In this study, the case of the criterion of the presence of wattles, which are appreciated, may be of particular interest in the framework of the conservation of the Koundoum breed, since this is a frequent characteristic of the breed (Hamadou et al., 2015a). The presence of wattles is thought to be a sign of good dairy aptitude in ewes (Meyer et al., 2004). Indeed, Casu et al. (1970) showed in Sardinian sheep in Italy that the presence of wattles in ewes coincided with productive superiority (prolificacy and milk production).

An important criterion that has been absent from focus group discussion is resistance or adaptation to the environment. Indeed, the adaptation of the Koundoum to its environment is its main advantage. As breeders recognise this resistance of Koundoum sheep, its absence from the cited criteria signals the lack of interest in the breed, tied to the overall change in the production environment and practices in the region (Hamadou et al., 2015a). The present results are also in line with the findings of resistance by Ibrahim (1998) on a list with examples of traits that are most often used as a basis for selection in small ruminants.

### 4.3 Preference for attributes of breeding rams

The negative sign of the coefficient associated with the price in the results of the conditional logit is in agreement with the expected disutility of expense and allows using this coefficient for the calculation of willingness to pay or willingness to accept compensation (Banerjee et al., 2006). However, some utility of expense may commonly result from the interpretation of price as a sign of quality (Siddo et al., 2015). Open questions at the end of each interview allowed us to dismiss this possibility as a main bias in this study. The most appreciated rams appear to be rams with long ears, bristles and large body size. While the preference for white-coloured rams could not be shown statistically, the strong dislike of black-coloured rams appears clearly. These preferences work against the conservation of the Koundoum breed, as developed here under, underlining the strong overall move towards the abandonment of this breed.

The particular importance of the long ears in the decision making of breeders is remarkable, as shown through a willingness to pay and its relative importance in decision making. This relative importance contrasts with the weight attributed by breeders to this same criterion through proportional piling. Also, the body size, which may be expected to be of major importance in systems aiming at the production of meat, displayed an astonishingly weak willingness to pay and relative importance in the decision making. In fact, from unpublished data not shown in the results, traditional farmers interpret long ears as a sign of the rapid growth of a ram. This belief may have led them to choose systematically all profiles showing long ears even in rams of smaller size.

The preference for bristle coats and thus the relative dislike of wool is a strong sign of the ongoing neglect of Koundoum sheep. The loss of value of wool in the region due to the lack of transformation and markets may have driven this loss of interest for the Koundoum breed (Hamadou et al., 2015a). Nevertheless, a lack of interest does not necessarily result in such a strong dislike of this precise attribute as observed in the present study. In this regard, Landais (1990) proposes another motivation for this dislike of wool, which is the abundance of pastures of grass with prickly seeds (*Cenchrus catharticus*) that invade the wool of Koundoum sheep. The preference for larger body sizes constitutes another unfavourable factor for Koundoum sheep, which is a rather small-sized breed (Hamadou et al., 2015a). Finally, black coats are frequent in Koundoum (Hamadou et al., 2015a). Let us note that the black colour of the Karakul sheep has also been a reason for the failure of its diffusion in Niger as farmers consider the black sheep to be cursed (Landais, 1990). Again, this observation highlights the importance of taking account of the objectives, preferences, constraints and beliefs of the breeders in animal genetic resource management.

Contrary to the wool attribute, body size and coat colour may be changed through selective breeding. Nevertheless, a breeding programme does not appear in the present case as a promising solution. Indeed, besides the fact that the breeders would have to be convinced to participate in such a demanding programme, this solution would involve special follow-up of the resistance criteria in the breeding process. Also, the low population presently available in the region entails a risk of a rapid rise in consanguinity. Finally, the overall loss of genetic diversity linked to the abandonment of black Koundoum sheep sharply contradicts the present goal of diversity conservation.

Thus, the finding that emerges is the systematic rejection of all the typical characteristics of Koundoum sheep by breeders with the exception of white coat colour. This rejection is more pronounced for the wool coat which is a typical characteristic of Koundoum sheep as demonstrated by the amount of EUR 226 as a willingness to receive compensation for keeping a wool ram. This amount represents around three times the greatest ram prices used in the experiment. A similar case was reported by Roessler et al. (2008) in Vietnam, where they found a very high willingness to pay for a “pig that rarely falls ill”, i.e. VND 40 000, while the greatest pig prices in their experiment is VND 28 000. In this study, rejection is also great for the black coat, for which breeders are willing to receive compensation of EUR 172 to keep the rams with this coat, i.e. 2.5 times the highest price considered in this study.

In general, any conservation programme involves a wide variety of stakeholders, who will be required to cooperate in the conservation of a breed and thus to make collective choices (Lauvie et al., 2008).

For this, it is necessary to understand the preferences of farmers regarding their domestic animal genetic resource management decisions and to consider appropriate criteria within the framework of conservation. Indeed, this study of preferences provides, in turn, favourable indications for the success of programmes for the conservation and sustainable management of a breed (Pattison et al., 2007).

According to Planchenault and Boutonnet (1997) the most common way of conserving genetic resources is to help farmers develop and use their breeds (in situ conservation). However, the finding of the present study is not in favour of this statement. While it is entirely consistent with the claim that the importance of livestock biodiversity may be critical to poor smallholders, for many other players the value of farm animal biodiversity will be a value option, i.e. a non-use value often running contrary to their short-term interests (Hamadou et al., 2016). In addition, at the national level, governments need data on the economic values of breeds and their characteristics in the development of incentive systems for in situ conservation programmes for these breeds (Scarpa et al., 2003). Thus, by showing the low feasibility of in situ conservation, the results of the study show the decision maker the need to focus on ex situ conservation in the framework of the conservation of Koundoum sheep. Nevertheless, a micro in situ conservation programme based on the few producers exclusively raising Koundoum sheep (Hamadou et al., 2015a) is conceivable.

The importance of biodiversity conservation for this decision maker is shown by the statement according to which the conservation and sustainable use of biological diversity creates opportunities to reduce poverty and improve human wellbeing and hence economic and human development (SCDB, 2009).

Therefore, the lack of appropriate measures for the conservation of livestock biodiversity is a serious concern, more especially as genetic erosion will cause losses that will have important impacts on the future socio-economic functions of livestock (FAO, 2008). Fortunately, in the past 2 decades, livestock diversity conservation has received international attention, being promoted as an opportunity to meet future and current market needs for food in the contexts of the diversification and evolution of productions (Shrestha et al., 2010). Animal genetic resources are described by Rege and Gibson (2003) as vital for the economic development of most countries in the world, playing an important role in the livelihoods of many communities in developing countries.

Moreover, if we try to broaden the debate on the implications of the methodology used in this study, some authors including Jabbar and Diedhiou (2003) argue that ex ante assessment of farmers' breeding strategies and breed preferences and market values of different breeds can assist breed conservation and improvement efforts in several ways. First, it can help to assess current stocks of different breeds held by farmers, their geographic distribution and the likely future trends. Interbreeding is more likely among animals raised in close proximity (as in the case of Koundoum sheep and Fulani sheep) and when different breeds are raised in the same herd.

Second, farmers' knowledge about specific attributes of different breeds under village conditions can help to focus scientific research on particular traits and identify needs for further education of farmers through extension programmes. The relationship between the length of the ears and the growth of sheep revealed by this study deserves special investigation in this species. Similar investigation was carried out by Casu et al. (1970) who associated the presence of wattles with a good ability to produce milk and prolificacy in the Sardinian dairy sheep breed in Sardinia. Third, it can help to determine the incentives that may need to be put in place for farmers to be involved in the conservation of threatened or endangered breeds that may not be supported by market forces. Fourth, information about farmers' breeding practices and breed preferences can help to identify the likely market for existing or improved breeds, as market information reveals buyer preferences for different breeds and attributes, which may be useful in the design of breed improvement schemes. Another information of general importance that deserves discussion is the positive willingness to pay for the white coat in contrast with the black colour. This result shows that the colour of the coat is an important criterion to be taken into account by sheep breeders, especially in Muslim countries. Indeed, according to Brisebarre and Kuczynski (2009), the “ideal sheep of Tabaski” must be a large, robust ram with well-developed horns and a white coat.

## 5 Conclusion

The present study was conducted to investigate the preferences of breeders within the framework of the conservation of Koundoum sheep in Niger. It represents the first use of the stated preference methods for the valuation of animal genetic resources in a conservation goal in Niger. The results reveal the strong rejection by breeders of characteristics such as wool and a black coat. The latter being typical characteristics of Koundoum sheep, these results indicate a lack of feasibility of in situ conservation programmes for this breed in this area. A few breeders with a cultural concern for the preservation of the breed may nevertheless join a conservation programme that should be mainly based on ex situ strategies.

## Supplement

10.5194/aab-62-537-2019-supplement The supplement related to this article is available online at: https://doi.org/10.5194/aab-62-537-2019-supplement.

## Data Availability

Readers can request the raw data from the corresponding author.
