# Supplementary material for: Valuing breeders' preferences in the conservation of the Koundoum sheep in Niger by multi-attribute analysis
Source: Arch Anim Breed. 2019 Sep 9;62(2):537–45. doi: 10.5194/aab-62-537-2019 (PMC6853032; doi:10.5194/aab-62-537-2019)
Supplement: The supplement related to this article is available online at: https://doi.org/10.5194/aab-62-537-2019-supplement. [file aab-62-537-supplement.pdf]

Supplement of Arch. Anim. Breed., 62, 537–545, 2019
https://doi.org/10.5194/aab-62-537-2019-supplement
© Author(s) 2019. This work is distributed under
the Creative Commons Attribution 4.0 License.

Open Access
Archives Animal Breeding

Supplement of

Valuing breeders' preferences in the conservation of the Koundoum sheep in Niger by multi-attribute analysis

Issa Hamadou et al.

Correspondence to: Nicolas Antoine-Moussiaux (nantoine@uliege.be)

The copyright of individual parts of the supplement might differ from the CC BY 4.0 License.

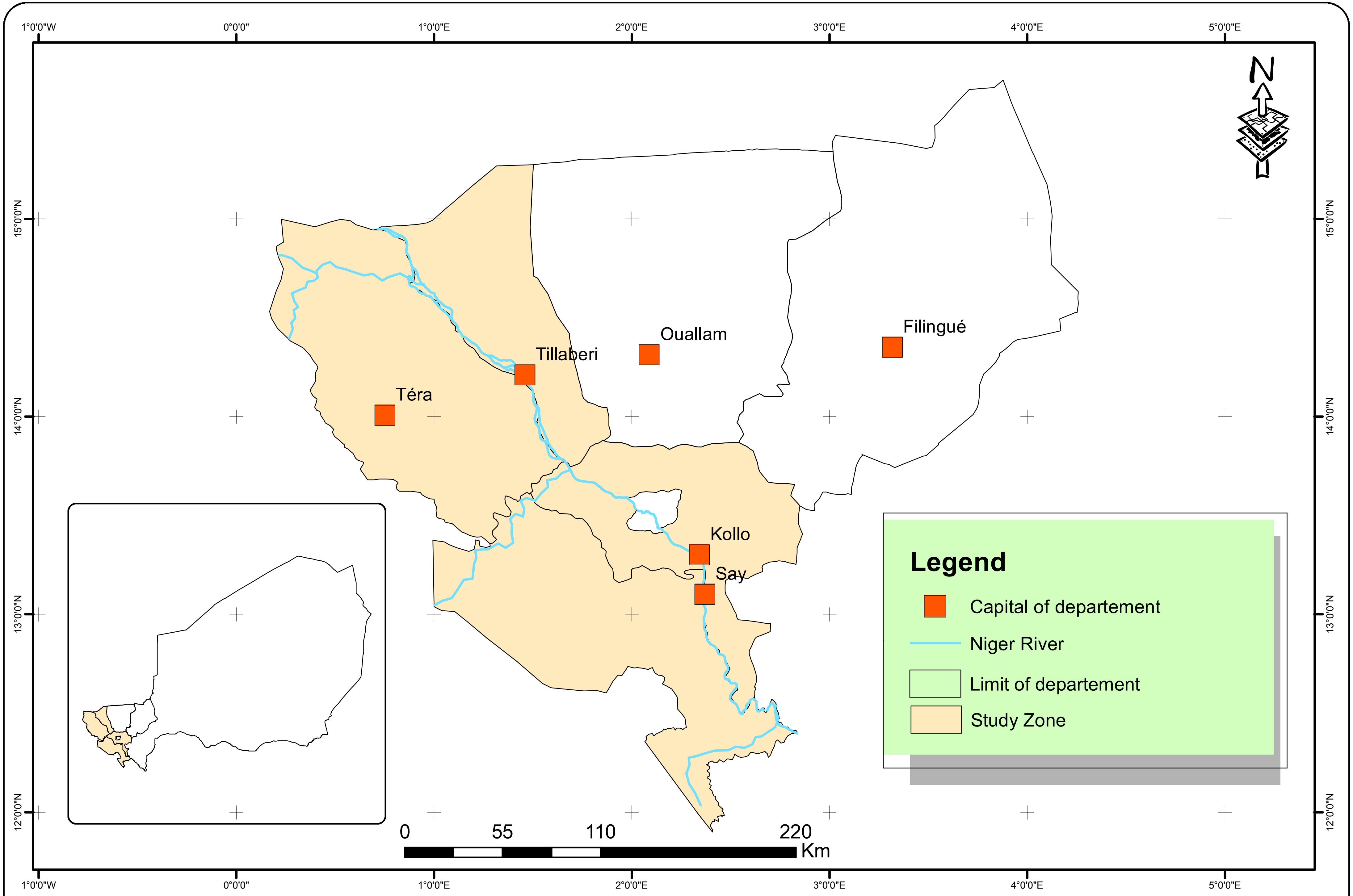

Coordinate System: GCS WGS 1984
Datum: WGS 1984
Units: Degree

©Labo-SIG INRAN, Mars 2019, Realization : Abdou M.A Djibrilla & I. Hamadou

Source : Database IGNN
